# Tracking of Mammals and Their Fleas for Plague Surveillance in Madagascar, 2018–2019

**DOI:** 10.4269/ajtmh.21-0974

**Published:** 2022-04-18

**Authors:** Soanandrasana Rahelinirina, Mireille Harimalala, Jerry Rakotoniaina, Mamy Gabriel Randriamanantsoa, Catherine Dentinger, Sarah Zohdy, Romain Girod, Minoarisoa Rajerison

**Affiliations:** ^1^ Plague Unit, Institut Pasteur de Madagascar, Antananarivo, Madagascar;; ^2^ Medical Entomology Unit, Institut Pasteur de Madagascar, Antananarivo, Madagascar;; ^3^ Central Laboratory for Plague, Ministry of Public Health, Antananarivo, Madagascar;; ^4^ National Plague Control Program, Ministry of Public Health, Antananarivo, Madagascar;; ^5^ U.S. President’s Malaria Initiative, Centers for Disease Control and Prevention, Antananarivo, Madagascar;; ^6^ U.S. President’s Malaria Initiative, Centers for Disease Control and Prevention, Atlanta, Georgia

## Abstract

Plague, a zoonotic disease caused by the bacterium *Yersinia pestis*, remains a major public health threat in Madagascar. To better understand the risk of transmission to humans and to guide targeted plague prevention and control measures, a survey of *Y. pestis* infection and exposure in mammals and their fleas was implemented. Small mammals were captured in five districts of Madagascar ranging in levels of plague endemicity, as measured by notified cases, from none to active foci. Blood and spleen samples and fleas were collected from small mammals for the detection of anti–*Y. pestis* F1 antibodies by ELISA, F1 antigens by rapid diagnostic tests, and *pla*, *caf1*, and *inv* genes by polymerase chain reaction. Some rodent fleas were kept alive and reared in the insectary to assess susceptibility to insecticides. Blood was also collected from 15 dogs and tested for anti-F1 antibodies. A total of 557 spleens, 484 sera, and 1,539 fleas were collected from 557 rodents and shrews. Nineteen (3.4%) spleens were positive for F1 antigen, most from Toamasina (*N* = 13), a historical plague focus. One dog was also found seropositive in Toamasina. Twenty-two (4.5%) serologic specimens from small mammals were positive for anti-F1 antibodies. The flea index was highest in the city of Antananarivo (8.8). No flea was positive for *Y. pestis* DNA. Flea populations exhibited resistance to various insecticides weakening the efficacy of vector control. This study highlights the potential use of animal-based surveillance to identify the risk of plague transmission in endemic and nonendemic foci for targeted prevention and control.

## INTRODUCTION

Plague, a zoonotic vector-borne disease caused by the bacterium *Yersinia pestis*, presents a significant public health challenge in Madagascar. From 2013 to 2018, an average of 387 human plague cases were reported annually, which is the highest national incidence in the world.^1^ Most of these cases were bubonic plague resulting from *Y. pestis* transmission during bites of infected fleas. However, in 2017, a pneumonic plague outbreak with human-to-human transmission occurred in the urban areas of Antananarivo and Toamasina.^2^ Human plague cases are often preceded by epizootics when large numbers of susceptible rodents die of infection and fleas seek human hosts.^3^ Thus, conducting surveillance for *Y. pestis* circulation in animals and fleas could be useful for monitoring the risk of plague transmission to humans.

Plague is endemic in Madagascar, particularly in the Central Highlands where the altitude is above 800 meters. Two rodent species, the black rat (*Rattus rattus*) and the brown rat (*Rattus norvegicus*), and one Asian house shrew (*Suncus murinus*) are invasive species and reservoirs from which *Y. pestis* can develop and be transmitted to another host.^4^^,^^5^ *Rattus rattus* is a commensal and sylvatic rat and is the primary plague reservoir in the rural foci where plague occurs most frequently in Madagascar^6^ while *R. norvegicus* is found in urban areas. Two flea species are known to play a role in the transmission of *Y. pestis* in Madagascar: the oriental rat flea (*Xenopsylla cheopis*) which is found in many parts of the world and the endemic flea (*Synopsyllus fonquerniei*) that is mainly present in the Central Highlands.^6^ The human flea (*Pulex irritans*), which has previously been suspected to play a role in human-to-human *Y. pestis* transmission in Europe^7^ and in Africa,^8^ is not considered a plague vector in Madagascar although *Y. pestis* DNA has previously been detected in some specimens.^9^

Plague diagnosis in human and animal samples (bubo aspirate, sputum, blood and organ) can be done by rapid diagnostic test (RDT) using a dipstick for Fraction 1 capsular antigen (F1) detection, by bacterial culture using selective culture medium, by ELISA that detects anti-F1 IgM and IgG, and by polymerase chain reaction (PCR) targeting *pla*, *caf1*, and *invasin (inv)* genes of *Y. pestis* in DNA extracts.^10^^–^^12^ In Madagascar, *Y. pestis* has been isolated from small mammals and fleas, and IgG antibodies have been detected approximately seven days post-infection in rats and shrews.^5^ IgG antibodies, indicating exposure to *Y. pestis*, can also be detected in domestic dogs (*Canis familiaris*).^14^ Therefore, these animals could serve as sentinel species for plague surveillance to better understand the eco-epidemiology of the disease and to contribute to public health strategies at local, national, and international levels.

During plague outbreaks, the use of insecticides to control flea populations is the primary response intervention. Therefore, monitoring flea susceptibility to these products is of major importance. In some rural and urban areas, *X. cheopis*, the main plague vector, has been reported to be resistant to pyrethroid and carbamate insecticides.^15^^,^^16^ More recently, resistance of *X. cheopis* to organophosphate insecticides has also been detected in some specific situations.^17^

The National Plague Control Program (NPCP) conducts routine surveillance for plague in humans, investigating all reported suspected cases, but no surveillance program in animals is conducted. To characterize the ecology of *Y. pestis* infection in endemic areas of Madagascar, estimate human risks, and guide targeted prevention and control measures, this study aimed to 1) assess *Y. pestis* infection and exposure in small mammal reservoirs and their fleas, 2) determine the proportion of domestic dogs with antiplague antibodies, and 3) evaluate the susceptibility of wild-caught flea populations to various insecticides.

## MATERIALS AND METHODS

### Study sites and sampling period.

**Figure 1.**
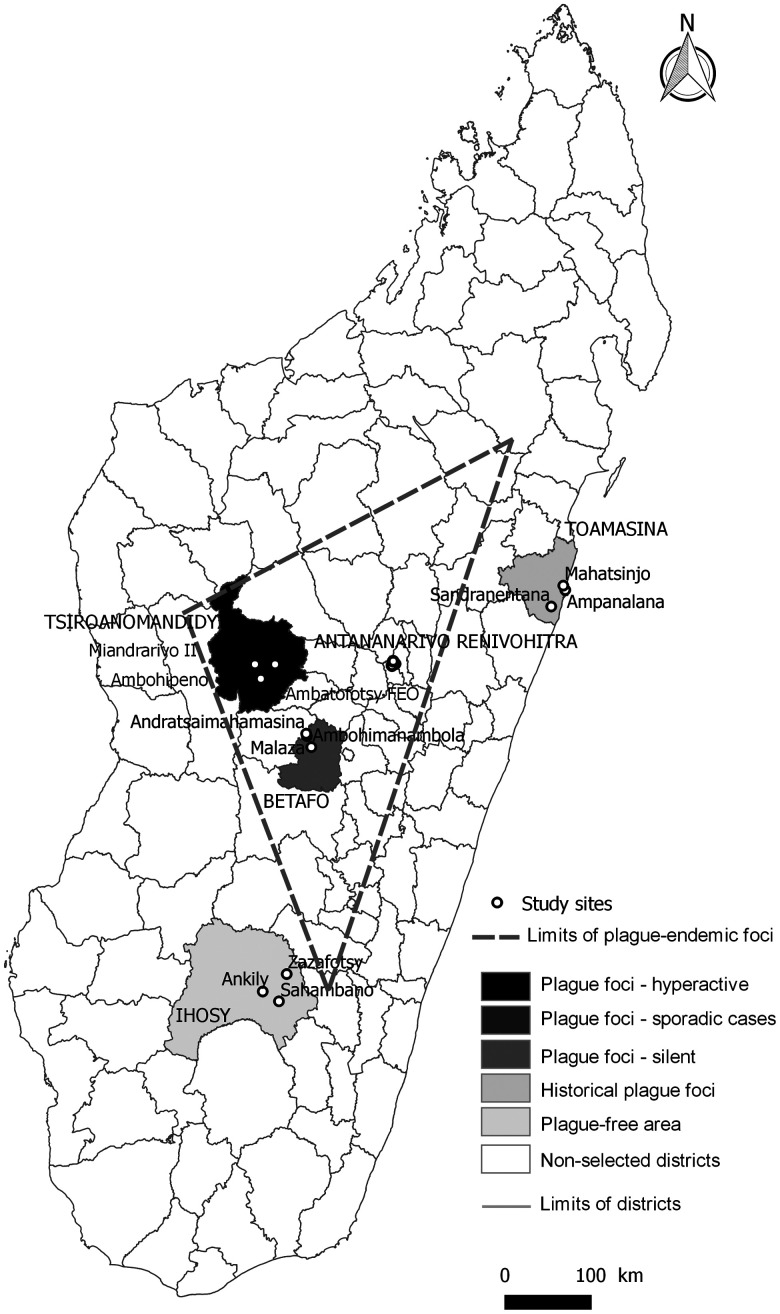
Map of Madagascar showing the study sites ranging by plague endemicity levels.

Sampling was conducted from December 2018 to May 2019 in 15 sites belonging to five districts ranging in plague endemicity levels from none to active foci (Figure 1, Supplemental Table 1). The five chosen districts were: Tsiroanomandidy, an active plague focus that has had confirmed human cases every year over the past decade; Betafo, a latent focus with no report of human plague between October 2015 and October 2018; Ihosy, an area where plague cases have never been detected; Toamasina, an historic focus where plague was introduced from infected rats on ships docking in the port in 1898 and where a pneumonic plague outbreak occurred in 2017; and Antananarivo Renivohitra, the capital city of Madagascar, which typically has sporadic cases but during 2017 was one of the outbreak focus.^2^

### Animal sampling and flea collection.

Small mammals were live-trapped using aluminum Sherman traps (H.B. Sherman Trap, Inc., Tallahassee, FL; 23 cm long × 7.5 cm wide × 9 cm high) for mice and shrews and wire-mesh BTS traps (Besancon Technical Service, Besancon, France; 30 cm long × 10 cm wide × 10 cm high) for rats. A total of 120 traps were placed inside 60 houses per village (one of each trap type per house). In addition, one line transect per village was set up outdoors (within a 200-meter radius) with 20 BTS traps set spaced 10 meters apart. In the urban sites of the district of Antananarivo Renivohitra, traps were set only inside houses. All traps were baited with dried fish and onion slices and set for three consecutive trapping nights per site. Captured small mammals were euthanized by cervical dislocation, and adult fleas were removed by fur brushing inside a basin of 30 cm depth, deep enough to prevent rat fleas from jumping out. Fleas escaping from the host were collected using a homemade flea vacuum,^18^ and counted to determine the flea index (the average number of fleas per host). Fleas collected per site were divided into two batches, fleas of the first batch (collected during the first and second nights) were kept alive in clear two-liter glass jars containing sterilized rice bran and larval food for insecticide testing and fleas of the second batch (collected during the third night) were stored in 1.5 mL microtubes containing 70% ethanol for *Y. pestis* DNA detection. The mammal hosts were identified, weighed, measured, and sex identified. Blood samples were collected by cardiac puncture and dropped onto serobuvard filter paper disks (LDA22, Zoopole, Ploufragan, France) and preserved at 4°C for serological analysis. Spleen samples were aseptically collected and conserved in Cary Blair medium, which was prepared at Institut Pasteur de Madagascar (IPM) and transported at ambient temperature to IPM’s Central Laboratory of Plague (CLP), for bacteriological analysis.

Domestic dwelling fleas were collected for 1 night in each of the sites using light traps^19^ consisting of a candle fixed in the middle of a plate containing soapy water that prevents fleas from escaping. Household residents lit the candle at night and left it burning for about 8 hours. Trapped fleas were collected using featherweight forceps, counted, and preserved in 70% ethanol for species identification and *Y. pestis* DNA detection.

Both small mammal fleas and domestic dwelling fleas were morphologically identified once in the IPM laboratory using a binocular magnifier and Harimalala’s keys.^20^

To assess the possibility of domestic dogs serving as sentinel species, saphenous vein blood was collected from selected domestic dogs less than 6 months old in all study sites. Sera were preserved at 4°C in the field and serologic assays were performed immediately upon arrival to the CLP to assess for exposure to *Y. pestis*.

### Ethical considerations.

All animals were captured and handled in accordance with guidelines of the American Society of Mammalogists,^21^ and the directive 2010/63/EU of the European Parliament (http://eur-lex.europa.eu/Lex-UriServ/LexUriServ.do?uri=OJ:L:2010:276:0033:0079:EN:PDF).

Field sampling was carried out systematically by joint teams of staff from IPM and the Ministry of Public Health. Each trapping campaign was validated by the national, regional, and local health authorities. The small mammals sampled were those from introduced species (house mouse, black rat, brown rat, and Asian house shrew), all of which are considered as pests, with no protected status in Madagascar.

Verbal authorization from the community’s authority and village head, verbal consent from the house or dog owners were also required after informing them of the objectives of the project.

### Detection of *Y. pestis* circulation.

Sera from small mammals and dogs were tested for IgG antibodies specific to the F1 of *Y. pestis* using a well-established ELISA.^22^^,^^23^ Spleen samples from small mammals were tested by RDT for *Y. pestis* F1 antigen,^11^ and positive samples were confirmed using bacterial culture.

For detecting of *Y. pestis* DNA from fleas, specimens were dried individually and crushed with a Tissue Lyser (Qiagen, Germany) using 3-mm tungsten beads and 75 µL of 1× phosphate-buffered saline. DNA extraction was performed using the DNA Blood and Tissue kit (Qiagen) following the manufacturer’s instructions. Detection of *Y. pestis* was examined by PCR targeting the plasminogen activator (*pla*), the F1 capsular antigen (*caf1*) and the *inv* genes. Primer sequences used were Yp1 (5'-ATCTTACTTTC CGTGAGAAG-3') and Yp2 (5'-CTTGGATGTTGAGCTTCCTA-3') for *pla*,^24^ Inv839 (5'-TACCTGCACTCCCACAAC-3') and Inv1007 (5'-CCCATACGCTGATCTACC-3') for inv,^25^ and, caf1-f (5'-ATAC TGCAGATGAAAAAAATCAGTTCC-3') and caf1-r (5'-ATAAAGC TTTTATTGGTTAGATACGGT-3') for *caf*.^26^ The preparation of the PCR mix, the thermal cycling conditions chosen for amplification using each pair of primers and the migration process of PCR products were used as reported by Harimalala et al.^27^

### Susceptibility of fleas to insecticides.

Live fleas preserved in glass jars were transported to IPM’s Medical Entomology Unit for further handling. To carry out morphological identification, fleas were collected, immobilized into a microtube placed on ice for 2 to 3 minutes and then transferred to a glass petri dish for observation under a binocular magnifier. Identification was done using Harimalala’s keys.^20^ Once identified, *X. cheopis* specimens were put back inside glass jars and reared under controlled conditions (temperature 25 ± 2°C and relative humidity 75% ± 5%). Insecticide susceptibility tests were performed on fleas of the next generations (first and/or second generations depending on the sample size) following the WHO protocol.^28^ Sixty fleas per test session were used corresponding to four replicates of 10 fleas exposed to insecticide and two replicates of 10 fleas serving as controls. Susceptibility of fleas to three insecticides was assessed: fenitrothion (organophosphate), which is currently used during outbreak responses; deltamethrin (pyrethroid), which was previously used; and permethrin (pyrethroid), which may be an alternative to fenitrothion. The concentrations of active substance used in the resistance assays (diagnostic doses) were determined according to WHO guidelines.^28^ The diagnostic dose was twice the lethal dose. The lethal dose was the lowest concentration at which 100% mortality of a laboratory strain susceptible to the tested insecticide was induced. Fleas were exposed to insecticide-impregnated papers (1.5 cm × 6 cm) in the following diagnostic concentration/duration combinations: fenitrothion 1% for 5 hours, and permethrin 0.75% and deltamethrin 0.05% for 8 hours each. After diagnostic exposure time, impregnated papers were removed and replaced by filter paper and tubes were left on the laboratory bench for 24 hours before recording the final mortality rate (MR). WHO guidelines^28^ were used to determine the susceptibility of fleas to insecticides. Flea populations were classified as resistant (MR < 80%), tolerant (80%≤ MR < 98%), or susceptible (98% ≤ MR ≤ 100%). If the MR of control fleas was over 20%, the test was considered invalid.

### Data analysis.

Data on captured animals and samples tested for *Y. pestis* were analyzed using descriptive statistics. Specifically, the abundance of small mammals per site was estimated from the overall trap success which is defined as the total number of individuals captured divided by the number of trap-nights, expressed as a percentage. Comparisons of percentages and means were performed using χ^2^ and Kruskal-Wallis tests and *P* values < 0.05 were considered statistically significant. Flea infestation per animal was calculated by taking the number of flea-infested small mammals divided by the total number of trapped small mammals. Rodent flea abundance per site was measured by calculating a flea index (FI) which is the number of fleas collected from small mammals divided by the total number of small mammals trapped (dead animals were excluded). Diversity and numbers of hosts and fleas collected and seroprevalence data were compared by using nonparametric tests with χ^2^ approximations. The statistical analyses were performed using R software, version 3.3.3 (R Core Team 2017).

## RESULTS

### Species abundance of small mammals.

From the 15 study sites, a total of 557 small mammals were captured belonging to three invasive rodent species: *R. rattus* (59.6%), *Mus musculus* (27.1%), and *R. norvegicus* (8.4%) and one invasive shrew species *S. murinus* (4.9%) (Table 1) more than 3,300 trap nights (16.9% trap success). *Rattus rattus* was the most captured species in rural areas compared with *R. norvegicus*, which was the most common in the capital Antananarivo. *Suncus murinus* was most captured in Toamasina. Only *R. rattus* and one *S. murinus* specimen were collected outdoor (Table 2).

**Table 1:** Distribution of small mammal species trapped in 15 study sites within five districts, Madagascar, December 2018–May 2019

Districts	Sites	No. captured animals	*Mus musculus*	*Rattus norvegicus*	*Rattus rattus*	*Suncus murinus*
Tsiroanomandidy (active plague focus)	Ambatofotsy FEO	72	5	0	65	2
	Ambohipeno	35	10	0	24	1
	Miandrarivo	59	16	0	39	4
	TOTAL (%)	166	31 (18.7%)	0	128 (77.1%)	7 (4.2%)
Antananarivo Renivohitra (plague focus with sporadic cases)	Ambanidia	28	9	16	0	3
	Anosizato Est	18	4	13	0	1
	Tsaramasay	17	4	13	0	0
	TOTAL (%)	63	17 (27.0%)	42 (66.7%)	0	4 (6.3%)
Betafo (latent focus)	Ambohimanambola	44	18	0	26	0
	Andratsaimahamasina	47	13	0	34	0
	Malaza	40	13	0	27	0
	TOTAL (%)	131	44 (33.6%)	0	87 (66.4%)	0
Ihosy (no plague human cases)	Ankily	43	17	0	26	0
	Sahambano	57	24	0	33	0
	Zazafotsy	31	14	0	17	0
	TOTAL (%)	131	55 (42.0%)	0	76 (58.0%)	0
Toamasina (historic focus)	Ampanalana	26	4	3	9	10
	Mahatsinjo	20	0	1	15	4
	Sandranentana	20	0	1	17	2
	Total (%)	66	4 (6.1%)	5 (7.6%)	41 (62.1%)	16 (24.2%)
Total (%)		557	151 (27.1%)	47 (8.4%)	332 (59.6%)	27 (4.9%)

**Table 2:** Plague risk indicators obtained from small mammal species trapped in 15 study sites within five districts, Madagascar, December 2018–May 2019

	Trap site	Small mammal species	No. captured	Fleas	FI	RDT positive no. (%) [95% CI]	Seropositive no. (%) [95% CI]
Tsiroanomandidy (active plague focus)	Indoor	MM	31	3	0.1	1 (3.2) [0.1–16.7]	0
		RR	113	219	1.9	2 (1.8) [0.2–6.2]	17 (15.0) [9.0–23.0]
		SM	7	12	1.7	0	0
	Outdoor	RR	15	61	4.1	0	1 (6.7) [0.2–31.9]
Antananarivo Renivohitra (plague focus with sporadic cases)	Indoor	MM	17	6	0.4	0	0
		RN	42	425	10.1	0	1 (2.4) [0.1–12.8]
		SM	4	1	0.3	0	0
Betafo (latent focus)	Indoor	MM	44	4	0.1	0	0
		RR	73	248	3.4	3 (4.1) [0.9–11.5]	0
	Outdoor	RR	14	5	0.4	0	0
Ihosy (no plague human cases)	Indoor	MM	55	14	0.3	0	0
		RR	72	477	6.6	0	0
	Outdoor	RR	4	1	0.3	0	0
Toamasina (historic plague focus)	Indoor	MM	4	0	0.0	0	0
		RN	5	38	7.6	0	0
		RR	41	24	0.4	3 (7.3) [1.5–19.9]	1 (2.4) [0.1–12.8]
		SM	15	1	0.1	9 (60.0) [32.3–83.7]	1 (6.7) [0.2–31.9]
	Outdoor	SM	1	0	0.0	1 (100.0) [2.5–100]	1 (100.0) [2.5–100]
Total			557	1,539	2.8	19 (3.4) [2.1–5.3]	22 (3.9) [2.5–5.9]

CI = confidence interval; FI = flea index; MM = *Mus musculus*; RDT = rapid diagnostic test; RR = *Rattus rattus*; RN = *Rattus norvegicus*; SM = *Suncus murinus.*

### Flea abundance.

Overall, 1,539 fleas were collected from small mammals and the majority were identified as *X. cheopis* (97.6%). Other species encountered were the sticktight flea *Echidnophaga gallinacea* (2.2%) and the cat flea *Ctenocephalides felis* (0.2%). The FI ranged from 0.1 to 8.8 in the different study sites, and the highest FI values were obtained from the district of Antananarivo Renivohitra followed by the district of Ihosy (FI = 6.3). Small mammals from the district of Toamasina had low flea infestations during this study (Table 3). All small mammal species were found to be flea infested; however, the house mouse (*M. musculus*) had no more than three fleas per animal (FI ≤ 0.4), whereas *R. norvegicus* was found to have up to 91 fleas per animal (Table 2).

**Table 3:** Plague risk indicators obtained from the 15 study sites within five districts, Madagascar, December 2018–May 2019

Districts	Sites	No. captured animals	Trap success	FI (no. flea)	Flea infestation no. (%) [95% CI]	Sero positive no. (%) [95% CI]	RDT positive no. (%) [95% CI]	Domestic dwelling fleas
Tsiroanomandidy (active plague focus)	Ambatofotsy FEO	72	30.0	2.7 (191)	48 (66.7) [54.6–77.3]	4 (5.6) [1.5–13.6]	2 (2.8) [0.3–9.7]	39
	Andranovelona	35	14.6	1.1 (39)	16 (45.7) [28.8–63.3]	3 (8.8) [1.9–23.7]	1 (2.9) [0.1–14.9]	37
	Miandrarivo	59	24.6	1.1 (65)	22 (37.3) [25.0–50.8]	11 (19.0) [9.9–31.4]	0	47
Antananarivo (plague focus with sporadic cases)	Ambanidia	28	15.6	5.0 (140)	16 (57.1) [37.2–75.5]	1 (3.6) [0.1–18.3]	0	23
	Anosizato est	18	10.0	7.9 (142)	12 (66.7) [41.0–86.7]	0	0	13
	Tsaramasay	17	9.4	8.8 (150)	14 (82.4) [56.6–96.2]	0	0	31
Betafo (latent focus)	Ambohimanambola	44	18.3	1.6 (71)	19 (43.2) [28.3–59.0]	0	3 (6.8) [1.4–18.7]	545
	Andratsaimahamasina	47	19.6	1.8 (86)	23 (48.9) [34.1–63.9]	0	0	489
	Malaza	40	16.7	2.5 (100)	18 (45.0.) [29.2–61.5]	0	0	94
Ihosy (no plague human cases)	Ankily	43	17.9	2.0 (86)	19 (44.2) [29.1–60.1]	0	0	17
	Sahambano	57	23.8	3.7 (212)	31 (54.4) [40.7–67.6]	0	0	287
	Zazafotsy	31	12.9	6.3 (194)	19 (61.3) [42.2–78.1]	0	0	216
Toamasina (historic plague focus)	Ampanalana	26	10.8	1.9 (50)	6 (23.1) [9.0–43.6]	1 (5.6) [0.1–27.3]	6 (23.1) [9.0–43.6]	0
	Mahatsinjo	20	8.3	0.1 (1)	4 (20.0) [5.7–43.7]	1 (5.0) [0.1–24.9]	5 (25.0) [8.7–49.1]	0
	Sandranentana	20	8.3	0.6 (12)	1 (5.0) [0.1–24.9]	0	2 (10.0) [1.2–31.7]	0

CI = confidence interval; FI = flea index; RDT = rapid diagnostic test.

A total of 1,838 domestic-dwelling fleas were collected from households using candle traps. The human flea (*P. irritan*s) was the predominant species collected (*N* = 1,772; 96.4%) although *C. felis* (*N* = 62; 3.4%) and *X. cheopis* (*N* = 4; 0.2%) were also collected. Flea numbers caught per house in one night ranged from 0 to 545, suggesting that domestic-dwelling flea abundance was heterogeneous among houses and sites; however, specific conditions affecting this distribution were not assessed. The highest flea numbers per house were found in study sites in the districts of Betafo and Ihosy (Table 3).

### *Y. pestis* infection.

Nineteen of 557 spleens of small mammals tested were positive for *Y. pestis* F1 antigen by RDT: three of 166 (1.8%) from Tsiroanomandidy (one from *M. musculus* and two from *R. rattus*), three of 131 (2.3%) from Betafo (all three from *R. rattus*), and 13 of 66 (19.7%) from Toamasina (three from *R. rattus* and 10 from *S. murinus*). Positive animals were trapped inside houses except one *S. murinus* from Toamasina (Table 2). No *Y. pestis* strain was isolated through bacterial culture.

Sera from 484 small mammals were tested, and 22 (4.5%) from seven sites within the districts of Tsiroanomandidy, Antananarivo Renivohitra, and Toamasina were positive for anti-F1 IgG antibodies. Rates of *Y. pestis* seropositivity were highest in Tsiroanomandidy (Table 2).

Of the 15 domestic dogs tested, only one (6.7%) was positive for anti-F1 IgG antibodies. This animal, aged ∼3 months, was from Toamasina.

No dead small mammal was observed in any site during the study.

A total of 1,332 flea samples from animals and 390 domestic-dwelling fleas from households were tested by PCR. All fleas were negative for *Y. pestis* DNA.

### Susceptibility of fleas to insecticides.

Flea populations from eleven sites within the five districts were tested to assess their susceptibility to three insecticides. The mortality rate (MR) was different among insecticides and populations. Among eleven populations tested with fenitrothion, eight were resistant (17.5 ≤ MR ≤ 67.5%), one was tolerant (MR = 97.5%) and two were susceptible (MR = 100%). All eight tested populations were resistant to permethrin (17.5% ≤ MR ≤ 62.5%). Six populations were resistant to deltamethrin (10.0% ≤ MR ≤ 42.5%) and one was tolerant (MR = 82.5%). In four sites of three districts (Ampanalana, Malaza, Ambatofotsy FEO, and Ambohipeno), resistance to all three insecticides was observed. Susceptibility to fenitrothion was found in two sites (Tsaramasay and Ambohimanambola) (Table 4). No flea control was undertaken before or during the study in the different study sites. Data on household use of insecticides for domestic insects like ants and cockroaches was not collected in this study.

**Table 4:** Mortality rates of fleas from 11 study sites within five districts, Madagascar, exposed to fenitrothion 1%, deltamethrin 0.05% and/or permethrin 0.75%

Districts	Sites	Fenitrothion, 1%	Permethrin, 0.75%	Deltamethrin, 0.05%
Tsiroanomandidy (active plague focus)	Ambatofotsy FEO indoor	67.5	61.8	32.5
	Ambatofotsy FEO outdoor	45.0	–	10.0
	Ambohipeno	57.5	35.0	15.0
	Miandrarivo	–	–	–
Antananarivo (plague focus with sporadic cases)	Ambanidia	97.5	20.0	82.5
	Anosizato est	–	50.0	25.0
	Tsaramasay	100.0	35.0	–
Betafo (latent focus)	Ambohimanambola	100.0	20.0	–
	Andratsay	–	–	–
	Malaza	62.5	62.5	42.5
Ihosy (no plague human cases)	Ankily	–	–	–
	Sahambano	20.0	–	–
	Zazafotsy	35.0	–	–
Toamasina (historic plague focus)	Ampanalana	17.5	17.5	20.0
	Mahatsinjo	–	–	–
	Sandranentana	52.5	–	–

## DISCUSSION

This is the first plague surveillance effort in Madagascar to identify public health risk indicators through *Y. pestis* infection and exposure in small mammals, domestic dogs, and host-associated and domestic-dwelling fleas across a range of sites with varying levels of plague endemicity. However, with only one or two alive small mammals captured per trap, it is possible that captures may have resulted in an underestimation of plague indicators including the possibility that healthy animals were trapped rather than sick ones. For this study, surveillance in healthy animals was also important as plague serology and *Y. pestis* carriage were both examined.

During this study, *Y. pestis* F1 antibodies and/or antigens were detected in all small mammal species surveyed: *M. musculus*,* R. norvegicus*,* R. rattus*, and* S. murinus*, but primarily in *R. rattus*. In rural areas *R. rattus* was the predominant small mammal species captured both indoors and outdoors, and *R. norvegicus* was most frequently detected in urban areas, as previously observed.^13^^,^^29^

*Suncus murinus*, an invasive shrew species and well-known reservoir for plague on the northwest coast of the island,^5^ represented 24.2% of small mammals captured in Toamasina, a district on the east coast of Madagascar where a pneumonic plague outbreak occurred in 2017.^2^ This study is the first to report the presence of *Y. pestis*–infected rodents (*R. rattus*) and shrews (*S. murinus*) in Toamasina where plague was first introduced to the island in 1898.^30^ The high prevalence of *Y. pestis* antigens in *S. murinus* indicated a very high level of exposure and suggests the potential role of this species as a natural reservoir in Toamasina. These results indicate that *S. murinus* may contribute to the maintenance of *Y. pestis* in this area. In Mahajanga in northwestern Madagascar, *S. murinus* which does not develop plague disease, is a known reservoir of *Y. pestis*.^5^ Further work will be useful to determine more in-depth *Y. pestis* and *S. murinus* dynamics in Toamasina. Recent studies demonstrated that *R. rattus* in Toamasina are susceptible to plague after *Y. pestis* infection^31^; however, the low abundance of fleas in this species may reduce the risk of transmission to humans. Because the bacterium can persist in soil for a long period,^32^^,^^33^ soil contamination may also act as a source for exposure of small mammals. *Suncus murinus* had the highest infection rate, and thus increased surveillance of the species and human cases in Toamasina, a historical plague area, is necessary to monitor *Y. pestis* strain circulation to avoid plague reemergence after a long absence. The work here focused on invasive species; however, there is often sympatry between invasive small mammals and native animals of conservation concern. It is possible that the detected circulation of *Y. pestis* in small mammals reported here, which has human health implications, may also influence wildlife conservation.

Dogs are known to prey on rodents and are generally considered to be suitable sentinel animals for a wide range of zoonotic pathogens due to their close associations with humans.^6^ Serological evidence of *Y. pestis* infection was noted in a 3-month-old domestic dog from the village of Ampanalana where the former quarantine hospital for plague (Lazaret) was located in Toamasina, indicating that the animal had been exposed to the bacteria. Carnivores can be infected with *Y. pestis* after eating infected rodents or being bitten by infected rodent fleas, but there is no evidence of dogs developing symptomatic plague, although they do seem to produce IgG antibodies to the bacteria.^14^^,^^34^ The presence of plague IgG antibodies in a young animal is an indication of recent exposure to *Y. pestis*.^34^ This could suggest that plague still circulates in this historical plague area, as supported by the presence of *S. murinus* and *R. rattus* positive for *Y. pestis* F1 antigen. Dogs have previously been reported to be seropositive for antiplague antibodies in Zimbabwe^35^ and the Democratic Republic of Congo (DRC).^36^ In Madagascar, the limited evidence suggests it is possible that dogs do not develop symptomatic plague, but in other countries, such as the United States^37^ (i.e., the state of New Mexico^38^), dogs have been shown to suffer from a mild febrile disease associated with infection. In Madagascar, the role of domestic dogs in the spread and maintenance of *Y. pestis* is still unknown.

In Tsiroanomandidy, a hyperendemic plague focus, 17 *R. rattus* were found positive for anti-F1 antigen IgG antibodies, and the detection of *Y. pestis* antigen in this species suggests active circulation of *Y. pestis*. In active plague areas, fleas may escape from infected rats to feed on available hosts including *M. musculus* of which 3.2% were found RDT positive. Previous studies showed that during the bubonic plague outbreaks in 2014 in Tsiroanomandidy, an investigation of small mammals showed that 8% of *R. rattus* were found to be RDT positive.^39^ Similarly, rodents were found to be RDT positive and seropositive in active plague foci in Tanzania,^40^^,^^41^ Zambia,^42^ and DRC.^36^ The percentage of animals that are RDT positive can be used to identify recent circulation of plague and may act as a sentinel warning sign for plague outbreaks.

In Antananarivo, the capital of Madagascar, one *R. norvegicus* (2.4%) was found seropositive. Between 1995 and 1997, up to 50% of rats were found seropositive and *Y. pestis* strains were isolated from *R. norvegicus* and *R. rattus*.^29^^,^^43^ Our results indicate that *Y. pestis* is still present and circulates at low rates in rat populations in Antananarivo. A previous study observed that *Rattus *spp. from Antananarivo do not develop plague despite *Y. pestis* exposure or infection,^44^ and the coexistence of resistant and susceptible animals in natural plague foci may contribute to the establishment and maintenance of *Y. pestis* in the district.

In Betafo, a district where no human cases had been reported in the previous 3 years, 2.3% of rodents were found to harbor *Y. pestis.* These results showed that plague still circulates in rodents in this latent focus, maintaining the enzootic cycle.

In Ihosy, a district where plague cases have never been detected, no small mammals tested positive for *Y. pestis*.

The majority of fleas collected from rats and shrews were *X. cheopis*, found across all 15 study sites. In the five districts, the FI varied from 0.1 to 8.8. It has been documented that an FI greater than 1.0 indicates potential risk for human plague transmission,^45^ but recently the number of *X. cheopis* per *R. rattus* to sustain plague transmission has been shown to be greater than 3.9 fleas per host.^46^^,^^47^ The substantive risks for plague are when competent fleas harbor plague bacterium and environmental conditions such as temperature and relative humidity are favorable for the development of fleas in all stages. Although *Y. pestis* circulates at low levels in small mammals in Antananarivo, the highest reported FI (8.8) was found here, indicating very high exposure risk to plague. The high FI in Antananarivo in this study is consistent with previous studies showing that FIs were 8.4 in 1999 and 6.5 in 2001.^29^^,^^43^ The second highest FI in this study was found in Ihosy, where the FI was 6.3, above the threshold of plague transmission. The use of the FI as surveillance indicator outside of the limits of endemic plague or in areas where plague has never been detected, may not seem intuitive; however, even in human plague-free areas, rats are generally susceptible to plague.^4^^,^^48^ Therefore, the high FI could enable transmission in case of introduction of *Y. pestis* in this area. During the time of this study, no *Y. pestis* was detected in fleas through PCR. Monitoring small mammals and fleas longitudinally may reveal trends about the abundance of fleas and seasonal trends in infestation rates.

The larger numbers of free-living human fleas *P. irritans* collected in human dwellings were observed in rural zones, especially in the districts of Betafo and Ihosy. The types of flooring materials likely have an impact on the biology of free-living fleas, particularly by fostering their larval development and adult emergence. In these rural zones, the flooring is often not made of cement as it is in other areas, such as in Antananarivo where numbers of *P. irritans* were not so high. *Pulex irritans* is a nuisance species for humans and may cause an allergic reaction, and its role as a plague vector is suspected in Madagascar.^9^ In Toamasina, fleas were absent in house dwellings. This may be due to the climate because this district is the rainy area of Madagascar and the wooden houses are built on stilts. Four *X. cheopis* were collected with light traps in Tsiroanomandidy and Ihosy. The presence of off-host rat fleas *X. cheopis* in human dwellings highlights the risk for human infection in rural areas where large numbers of people sleep on the floor. The nondetection of *Y. pestis* DNA in all domestic-dwelling fleas suggests that the chances of transmitting the bacterium from small mammals to humans via domestic-dwelling fleas was minimal at the time of sampling.

There may also be other factors associated with flea abundance that were outside of the scope of this study and therefore not adequately assessed. This study showed an inconsistency between *Y. pestis* antibody and antigen detection in small mammals and *Y. pestis* DNA detection in fleas. The detection of *Y. pestis* DNA in fleas appears to be a less sensitive surveillance method.

The resistance of arthropod vectors to insecticides is a global challenge due to the widespread use of insecticides. Most resistance studies show that the continuous and repeated application of the same class of insecticides creates high levels of resistance.^17^^,^^49^ In Madagascar, the NPCP has recommended the use of insecticides for decades to control flea populations in the event of plague cases. Fenitrothion (an organophosphate) in powder form is currently used indoors, but pyrethroids and carbamates have also been used for many years. It has also been proposed that the use of insecticides for mosquito vector control could be related to insecticide resistance in fleas.^50^ In Madagascar, the use of indoor residual spraying (IRS) for malaria control is implemented regularly by the National Malaria Control Program; however, no study has been conducted to assess the association between malaria IRS and susceptibility of the rat fleas. Some studies have also shown an association between the development of resistance in arthropod vectors and the application of agricultural pesticides.^51^ In this study, resistance or tolerance of *X. cheopis* to permethrin and deltamethrin was detected as observed previously.^15^^,^^16^ The use of pyrethroid-based insecticide formulations to control an and cockroach populations is a common practice throughout Madagascar, which also constitutes a credible hypothesis to explain the resistance profiles detected here. Some flea populations were found to be susceptible to fenitrothion in some sites, which is reassuring because this insecticide is used in response to plague outbreaks in rural and urban settings. However, the detection of fenitrothion-resistant or tolerant fleas in other sites, as more recently observed in some vulnerable communities,^17^ suggests the need for increased insecticide resistance monitoring in fleas to define the insecticide use and to implement strategies such as rotating insecticides to optimize flea control efforts. This situation of multi-insecticide resistance highlights the need to perform research to identify new insecticides with different mechanisms of action, new formulations, and new application methods such as the use of systemic insecticides^52^ that are effective at reducing flea populations in the field.^53^ In addition, insecticide-free methods for flea control are needed.

*Y. pestis* surveillance among small mammals should be proposed as a strategy to monitor bacteria circulation and activate targeted prevention and control measures. This study highlights the public health interest of ecological indicators such as presence of antibodies and *Y. pestis* carriage, flea index, and rodent diversity and presence for triggering targeted plague prevention and control measures. However, these ecological indicators, used independently or in combination, can suggest different responses. When *Y. pestis* is detected from small mammals or fleas by culture or PCR, even if FI is < 1, the Ministry of Public Health’s response plan is activated (live trapping of rodents, insecticide dusting, using of bait stations, and sensitization of the residents). Flea index is a poor predictor of plague activity if *Y. pestis* is not detected but should be used to inform vector control. Finding seropositive animals in the absence of any positive fleas or rodents by direct detection suggests either epizootic is no longer ongoing or that plague circulation occurred previously and insecticide application might not be needed, whereas direct detection might require a more immediate response. Due to the short study period, rodent density was not specifically calculated. Our findings reinforce existing recommendations in plague foci and highlight the need to continuously follow plague control measures to avoid exposure to infected rodents and their fleas. Our data also show that fenitrothion may still be a useful prevention tool in selected sites; however, the presence of resistance in other sites suggests that regular monitoring of the susceptibility of fleas to insecticides should be performed in all plague foci and the insecticides being used should be rotated to slow the development of insecticide resistance. These approaches will prolong the effective use of vector control tools and may be used to develop optimal plague vector control strategies for each location. The detection of *Y. pestis* in small mammal species in historic plague foci where plague is now considered extinct suggests that longitudinal monitoring in plague foci (including those which are not currently considered active), including during the low plague season (May–August), may be necessary for early detection of plague. Taken together, these strategies may be used to prevent and limit the public health impact of plague outbreaks in Madagascar.

## Supplemental Material


Supplemental materials
