# Supplementary material for: Tracking of Mammals and Their Fleas for Plague Surveillance in Madagascar, 2018–2019
Source: Am J Trop Med Hyg. 2022 Apr 18;106(6):1601–9. doi: 10.4269/ajtmh.21-0974 (PMC9209941; doi:10.4269/ajtmh.21-0974)
Supplement: Supplementary file 1 [file tpmd210974.SD1.pdf]

Supplementary file

Table: Description of study sites

District	Village or quartier	Latitude	Longitude	Altitude (m)	Zone	Trapping
**Tsiroanomandidy (Active plague focus)**	Ambatofotsy ferme	-18.93901	45.98706	800	Rural	Indoor/outdoor
	Ambohipeno	-18.93813	46.21001	892	Rural	Indoor /outdoor
	Miandrarivo	-19.0909	46.05315	790	Rural	Indoor /outdoor
**Antananarivo (Plague focus with sporadic cases)**	Ambanidia	-18.91941	47.53822	1318	Urban	Indoor
	Anosizato est	-18.93869	47.50452	1255	Urban	Indoor
	Tsaramasay	-18.89180	47.51708	1264	Urban	Indoor
**Betafo (Latent focus)**	Ambohimanambola	-19.69137	46.56835	1129	Rural	Indoor /outdoor
	Andratsay	-19.6628	46.56109	1179	Rural	Indoor /outdoor
	Malaza	-19.80772	46.61998	1317	Rural	Indoor /outdoor
**Ihosy (No plague human cases)**	Ankily	-22.48868	46.28218	698	Rural	Indoor /outdoor
	Sahambano	-22.38665	46.09756	726	Rural	Indoor /outdoor
	Zazafotsy	-22.2005	46.36723	764	Rural	Indoor /outdoor
**Toamasina (Historic plague focus)**	Ampanalana	-18.10766	49.39958	15	Rural	Indoor /outdoor
	Mahatsinjo	-18.28519	49.24996	23	Rural	Indoor /outdoor
	Sandranentana	-18.06232	49.37705	27	Rural	Indoor /outdoor
